# Temperature and moisture dependence of daily growth of Scots pine (*Pinus sylvestris* L.) roots in Southern Finland

**DOI:** 10.1093/treephys/tpz131

**Published:** 2019-12-20

**Authors:** Yiyang Ding, Pauliina Schiestl-Aalto, Heljä-Sisko Helmisaari, Naoki Makita, Kira Ryhti, Liisa Kulmala

**Affiliations:** 1 Department of Forest Sciences, University of Helsinki, PO Box 27, FI-00014 Helsinki, Finland; 2 Institute for Atmospheric Sciences and Earth System Research (INAR)/Forest sciences, University of Helsinki, PO Box 64, FI-00014 Helsinki, Finland; 3 Department of Forest Ecology and Management, Swedish University of Agricultural Sciences (SLU), Skogens ekologi och skötsel, 90183 Umeå, Sweden; 4 Faculty of Science, Shinshu University, 3-1-1 Asahi, Matsumoto-city, Nagano, Japan; 5 Finnish Meteorological Institute, PO Box 503, FI-00101 Helsinki, Finland

**Keywords:** belowground, climate change, drought, growth modeling, root phenology

## Abstract

Scots pine (*Pinus sylvestris* L.) is one of the most important conifers in Northern Europe. In boreal forests, over one-third of net primary production is allocated to roots. Pioneer roots expand the horizontal and vertical root systems and transport nutrients and water from belowground to aboveground. Fibrous roots, often colonized by mycorrhiza, emerge from the pioneer roots and absorb water and nutrients from the soil. In this study, we installed three flatbed scanners to detect the daily growth of both pioneer and fibrous roots of Scots pine during the growing season of 2018, a year with an unexpected summer drought in Southern Finland. The growth rate of both types of roots had a positive relationship with temperature. However, the relations between root elongation rate and soil moisture differed significantly between scanners and between root types indicating spatial heterogeneity in soil moisture. The pioneer roots were more tolerant to severe environmental conditions than the fibrous roots. The pioneer roots initiated elongation earlier and ceased it later than the fibrous roots. Elongation ended when the temperature dropped below the threshold temperature of 4 °C for pioneer roots and 6 °C for fibrous roots. During the summer drought, the fibrous roots halted root surface area growth at the beginning of the drought, but there was no drought effect on the pioneer roots over the same period. To compare the timing of root production and the aboveground organs’ production, we used the CASSIA model, which estimates the aboveground tree carbon dynamics. In this study, root growth started and ceased later than growth of aboveground organs. Pioneer roots accounted for 87% of total root productivity. We suggest that future carbon allocation models should separate the roots by root types (pioneer and fibrous), as their growth patterns are different and they have different reactions to changes in the soil environment.

## Introduction

Annual production of fine roots in boreal forests varies from 31 to 66% of total forest annual production (Kleja et al. 2008, Hansson et al. 2013a, Hansson et al. 2013b, Leppälammi-Kujansuu et al. 2014, Ding et al. 2019), which is higher than the global average of 22–30% (Jackson et al. 1997, McCormack et al. 2015a). In contrast to aboveground components, very little is known about fine root growth phenology (Steinaker and Wilson 2008) despite a large portion of belowground production (mainly on fine roots). Although short-term growth responses of the aboveground tree components to weather variables are well studied (Schiestl-Aalto et al. 2015), very few high temporal resolution studies on belowground phenology exist (Menzel et al. 2006, Cleland et al. 2007). In quantitative models, the pattern of root growth phenology has earlier been synchronized with shoot growth phenology (Krinner et al. 2005, Thornton and Zimmermann 2007, Oleson et al. 2010). However, recent evidence shows that root growth phenology is in asynchrony with the shoot phenology across a variety of biomes (Steinaker et al. 2010, Du and Fang 2014, Abramoff and Finzi 2015, McCormack et al. 2015b, Blume-Werry et al. 2016). Belowground root growth occurred simultaneously with aboveground growth in temperate forests, but in the north, root growth peaked ca. 50 days later than shoot growth (Abramoff and Finzi 2015). Correspondingly, root growth initiated, peaked and ceased later than leaf growth in both broad-leaved and coniferous boreal forests (Du and Fang 2014).

Measuring root growth directly is challenging, and thus, most root phenology studies have either observation time intervals of over 2 weeks or use an indirect observation method (e.g., soil respiration) to estimate root growth (Pregitzer et al. 2000, Du and Fang 2014, McCormack et al. 2014, McCormack et al. 2017). However, as belowground allocation is a major component in tree carbon budgets, it is important to understand the responses of root growth to short-term and long-term fluctuations in environmental factors as well. As climate warming changes the growing conditions of trees, understanding of these responses is crucial to predicting how whole tree growth and forest productivity will change in the future.

Temperature is the driving factor for the root extension rate when other factors, such as soil moisture and nutrients, are sufficient (Pregitzer et al. 2000, Steinaker et al. 2010, Du and Fang 2014, Delpierre et al. 2016). At timescales of weeks or more, temperature has also an indirect effect on the extension of roots. In boreal coniferous forests, the seasonal variations of temperature control photosynthetic phases and the whole tree productivity (Kramer et al. 2000). Global warming was reported to exacerbate summer droughts on European soil, especially on boreal and continental regions (Samaniego et al. 2018). A severe drought occurred in Nordic countries in summer 2018 (World Meteorological Organization 2018). Soil moisture is typically highest in spring when snow melts and lowest in late summer when evaporation is greater than rainfall in Southern Finland (Hari and Kulmala 2008). The response of fine root biomass to drought varies by species. In Europe, it has been widely observed that conifers tend to increase root biomass, whereas the broad-leaved species tend to decrease root biomass simultaneously with decreasing soil moisture (Lukac and Godbold 2011). In humid conditions, negative correlation between root elongation and soil moisture may be caused by negative covariance between soil temperature and moisture (Steinaker et al. 2010). As an indirect effect, drought might advance the cessation of aboveground growth of Scots pine, causing the mobile carbohydrate pools to shift from aboveground to belowground for increasing root growth demands (Gruber et al. 2012).

Morphology of roots of perennial woody plants is heterogeneous. Fine roots, defined as the non-woody or absorptive roots that grow at distal positions of the root system, vary in physiology and longevity traits (Pregitzer et al. 2002). Absorptive roots have primary development mainly in the first three orders of roots, especially in the first order roots, while second and third order roots could have both primary and secondary development (Guo et al. 2008). Based on morphology differences, fine roots in woody plants are divided into pioneer and fibrous roots. Pioneer roots are so-called primary, long or skeletal roots; they are generally straight, thick in diameter, fast-growing and have prominent white tips at their distal parts. Fibrous roots are also called short, feeder or absorptive roots; they are relatively short and ephemeral compared to pioneer roots (Kolesnikov 1971, Lyford 1980, Sutton and Tinus 1983, Eissenstat and Achor 1999). Not only the growth rates and the external surfaces of the pioneer and fibrous roots are different, they also vary in physiological traits on the anatomical level (Bagniewska-Zadworna et al. 2014). In a root stele anatomy study, over 50% of pioneer roots of citrus cultivars in field experiments undergo secondary xylem development, whereas fibrous roots in the field rarely do (Eissenstat and Achor 1999). With secondary xylem development, pioneer roots have the ability to build the framework in the root structure (Zadworny and Eissenstat 2011). Due to their anatomical differences, pioneer roots are used for transporting and fibrous roots for nutrient and water absorption (Zadworny and Eissenstat 2011). In dry conditions, fibrous roots tend to have lower tissue density and lower suberin content than pioneer roots (Polverigiani et al. 2011). Because of the differences in both their structure and function, these two root types may also respond to environmental factors in different ways (Polverigiani et al. 2011).

The methods to determine root growth phenology have been limited. Unlike aboveground tree growth phenology, belowground phenology studies usually experience difficulties in observing the daily growth rate with indirect methods (e.g., root respiration), or with expensive and labor-intensive (e.g., minirhizotron (MR) and root window) direct methods. The MR method has been the most widely used method to measure fine root growth and turnover with minimal disturbance. However, the fast-growing pioneer roots can grow out of the screen in a few days due to the limited observation size (1.1 × 2 cm) of the MR image. Thus, it is not possible to trace the growth dynamics of pioneer root on a horizontal level over a longer period using MR method. The CI-600 cylindrical MR scanner system (CID Bio-Science, Inc.) and flatbed scanner system were developed to overcome this problem. The size of the observation area in these two types of scanners is the same. The flatbed scanner method is the most recently developed method to observe the root dynamics but is still rarely used (Dannoura et al. 2008, Dannoura et al. 2012, Nakano et al. 2012, Nakahata and Osawa 2017). Compared to manual methods (such as the MR and root window methods), the greatest advantage of the flatbed scanner method is that being connected to a computer, it can capture images automatically daily or hourly with little labor. In addition, the cost of installing and collecting the images with a scanner is 5–7% of that of the MR equipment (Dannoura et al. 2008).

We aimed to study the internal and environmental controls of Scots pine root growth under natural field conditions. Our main objective was to detect the relationships between soil environmental conditions (temperature and moisture) and the elongation rate of pioneer and fibrous roots. Furthermore, we compared the observed root elongation phenology with the growth phenology of aboveground components, i.e., shoots, needles, buds and secondary xylem. Growth of the aboveground components was estimated with the CASSIA model (Schiestl-Aalto et al. 2015) that has been developed and tested at the same research site.

We hypothesized that

1. Root elongation rate of Scots pine (*Pinus sylvestris* L.) has a positive relationship with soil temperature and moisture for both types of roots;
2. Fibrous roots are more sensitive to changes in the environmental conditions such as drought and low temperature than pioneer roots;
3. Root growth of Scots pine begins and ceases later than shoot growth.

To study these questions, we measured daily root growth with three flatbed scanners at an intensively monitored ecosystem station in Southern Finland and compared the results with simulated growth of aboveground parts.

**Figure 1.**
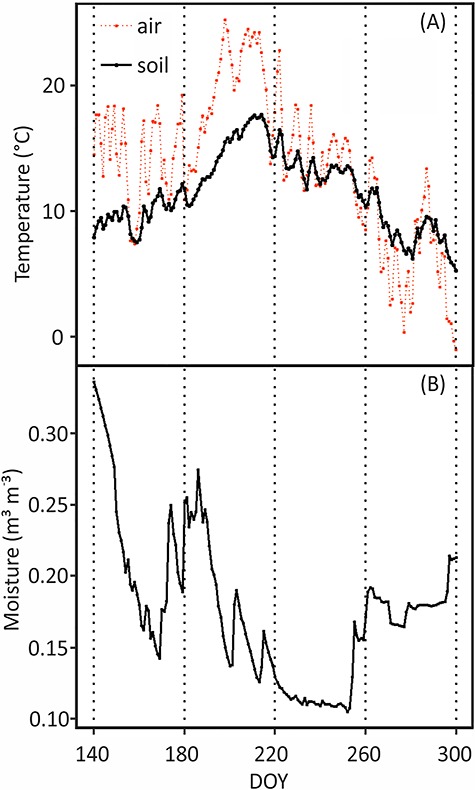
(A) Daily mean of soil temperature at depth of 2–5 cm, air temperature (°C) and (B) soil moisture (m^3^ m^−3^) at depth of 14–25 cm during the experimental period. Soil moisture indicates soil VWC. DOY stands for day of year, the sequential day number starting from 1 Jan 2018.

## Materials and methods

### Site characteristics

The study was conducted at the intensive research site at Hyytiälä SMEAR II (Station for Measuring Forest Ecosystem-Atmosphere Relations) of the University of Helsinki in Southern Finland (61°51 N, 24°17 E, 180 m above sea level). Our study site is a managed 56-year-old Scots pine dominated boreal forest mixed with Norway spruce (*Picea abies L. Karst*) and birch (*Betula spp.*) seedlings. The site was sowed after prescribed burning and minor soil preparation in 1962 (for more details, see Hari and Kulmala (2005)). The soil is classified as Haplic Podzol according to FAO-Unesco soil classification system (1997), and the mineral soil layer above the bedrock is only 0.5–0.7-m thick. The average organic layer thickness is 4.5 cm. The mean diameter of breast height of mature trees was 17.8 cm in 2016 (Schiestl-Aalto et al. 2019). The mean annual temperature (mean for 1980–2009) of the study site is +3.5 °C, and mean monthly temperature varies from −7.7 °C in February to 16 °C in July. The mean accumulated annual precipitation is 711 mm, the highest monthly accumulation being in July and lowest in February to April (Pirinen et al. 2012).

### Temperature and soil moisture

Meteorological parameters of continuous soil temperature, moisture and air temperature were obtained from the SmartSmear AVAA portal of the University of Helsinki Hyytiälä SMEAR II site (Junninen et al. 2009). Soil temperature (Figure 1A) was measured at the soil A-horizon (2–5 cm) with thermocouples at 15-min intervals. Soil moisture was measured as volumetric water content (VWC) with time-domain reflectometry at 15-min intervals in the B1 horizon (14–25 cm) since the water content sensor was broken in the A-horizon during the midsummer. We measured soil temperature by using thermocouples in the depth of 10 cm right at scanner surface and 15 cm away to reveal potential warming effects by the scanners. The temperature at the scanner surface was similar at nighttime and slightly higher during daytime compared with the soil temperature at 15 cm distance from the scanner, but the temperature difference was typically less than 1 °C. Therefore, we concluded that the soil temperature measurements around the site describe the temperature on the scanner surfaces accurately enough.

### Scanner method to observe root elongation

We installed three flatbed computer scanners into the soil to measure root elongation. Two out of the three scanners were Epson Perfection V39 and one was an Epson Perfection V37 (Seiko Epson, Tokyo, Japan). A schematic diagram of the installation procedure of the scanners is in Figure S1 (available as Supplementary Data at *Tree Physiology* Online). The scanners were protected with acrylic boxes (445 mm in length, 300 in height and 55 mm in width) to prevent water from entering the scanners (Figure S1A and B available as Supplementary Data at *Tree Physiology* Online). The three protection boxes were installed vertically into the soil, the long edge parallel with ground level. First, the organic layer was carefully removed and a rectangular hole for the box was made by a hand shovel, then the box was placed into the hole (Figure S1C and D). Thereafter, the remaining space was filled with root-free soil. In this procedure, we made sure that the soil was not too loose or compacted next to the scanner surface. For more installation details, see Kume et al. (2018). For Epson V39 scanners, we cut the side of the box next to the optics to fit the scanner glass area since this model cannot focus through the 5-mm-thick acryl screen. The cut edges were sealed with Sikaflex sealant (Oy Sika Finland Ab) to prevent moisture from getting inside the scanner or the box (Figure S1A). There was a removable cover on the acryl box enabling drying of the silica gel bag installed inside the box. The scanners were named Scanner 1 (Epson V37), Scanner 2 (Epson V39) and Scanner 3 (Epson V39). All the three scanners were connected with a USB cable to a personal computer (PC) in the nearby cottage. The computer controlled the images captured automatically using the free software NAPS2 (not another PDF Scanner 2). Scanners 2 and 3 were powered via the USB connection with the PC whereas a separate power line from the station powered Scanner 1.

**Figure 2.**
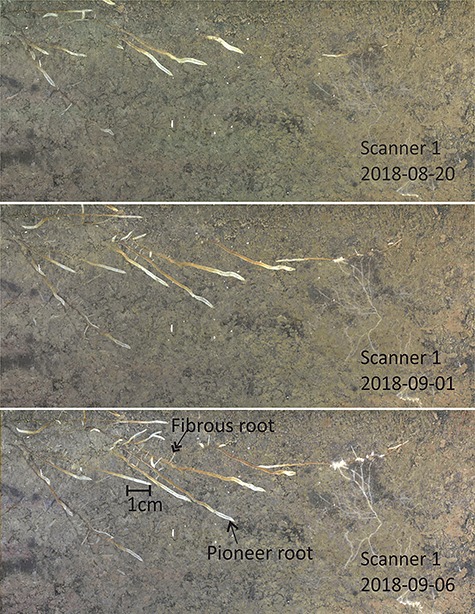
Time series images of scanner 1 on dates 20 Aug, 1 Sep and 6 Sep 2018. The images were cropped for better visual performance. The arrows indicate a pioneer root (single arrow) and a fibrous root (double arrow).

Scanners 1 and 3 were installed in April 2018 and Scanner 2 in May 2017. The scanners were randomly buried at approximately 1 m distance to the closest mature Scots pine trunk. Only mature Scots pine trees grew around the scanners whereas the nearest spruce seedling was at least 5 m away from any scanner. The images were captured once per day from the time they were installed (example images can be found in Figure 2). We only analyzed the images taken from the emergence of first roots until the root growth had ceased. During days of year (DOY) 140-303, there was visible root elongation on the screen. There were a few days with images missing due to connection and software problems: these dates were 27 May 2018–28 May 2018, 4 August 2018 (for Scanner 2 only); 29 August 2018–2 September 2018, 14 October 2018 (for Scanner 3 only).

### Image analysis

WinRHIZO TRON 2015a software (Regent Instruments Inc., Quebec, Canada) was used to analyze the collected scanner images. The scanner image size was 210 × 297 mm, as required by the software (for scanners). The diameter and length of each root were manually traced based on daily images. Besides these factors, information on root average depth, total average diameter, total length, total volume and total surface area were automatically documented by the software.

As roots with diameter <1 mm represent a highly heterogeneous component of root system consisting of both transport (pioneer) and absorptive (fibrous) roots (Pregitzer 2002), root structure and growth characteristics need to be reconsidered during the separation: pioneer roots extend relatively faster and have skeleton structures, whereas fibrous roots extend relatively more slowly and they often branch from the pioneer roots. The detailed criteria for distinguishing pioneer and fibrous roots are described by Kolesnikov (1971), Lyford (1980), Sutton and Tinus (1983), Eissenstat and Achor (1999). Indeed, all of the observed fibrous roots and 46% of pioneer roots in the study were under 1 mm in average root diameter.

In woody species (*Pinus spp.*, etc.), the ectomycorrhizal (EcM) colonization of lateral roots restricts the apical elongation and radial enlargement of the root caps, which means that EcM colonization may inhibit the elongation of first-order roots via IAA production from colonized fungi (Berta et al. 1990, Barker et al. 1998, Smith and Read 2010). In this study, the clusters of EcM infected most distal root tips (Helmisaari et al. 2009), which have a typical dichotomous root branching shape, and thus the root tips were not as visible as they would be in MR images. Thus, these clusters of EcM root tips were excluded from this study. We marked the time when the roots stopped elongation (i.e., they did not extend any more in subsequent images) in order to determine the growing period for each root. The birth time of pioneer roots may have some uncertainties since the pioneer roots may be born before they appear on the scanner screen. The birth time of the fibrous roots, which commonly branched from the pioneer roots, can be determined more precisely.

The roots that grew out of the scanner screen coverage during the observation period were excluded from all data analysis, because the elongation duration of these roots could not be determined. The first root appeared on Scanner 2 on 7 May (DOY 127), but later on, this root grew out of the observation area and was therefore excluded from further data analysis. The root initiation date of Scanner 2 on 7 May was still included in the root characteristics information. The root characteristics data such as root diameter, root length and root number were obtained from the final pictures (31 Oct, DOY 303). The growth of the total root surface area accumulated in the three scanners during the growing season was used to reflect the actual production of the pioneer and fibrous roots.

## Statistical analysis

The mean daily growth rate of pioneer and fibrous roots was calculated separately for each scanner. The daily root growth rate was calculated as the accumulated daily elongation of roots divided by the active root number i.e., the number of elongating roots. A two-way ANOVA was conducted to assess the differences in the root characteristics such as root diameter (RDiam), root length (RL), growing period (GP) and the differences in the growth between root types (pioneer and fibrous) and between scanners (Scanner 1, Scanner 2 and Scanner 3), followed by Tukey’s post hoc test to determine the specific differences between the groups (*P* < 0.05). The collinearity of covariates was checked by the variance inflation factor (VIF < 3) (Zuur et al. 2010). The outliers of the growth rate (>4 mm d−1) were excluded.

We built linear mixed-effect models to test the effects of soil temperature (*T*), soil moisture (*M*), root types (*R*) and spatial variability (*S*) on daily root elongation rate (mm day^−1^). In practice, the three different scanners were used as indicators of possible spatial variability. Root type gets a value 0 or 1 for fibrous and pioneer roots, respectively. Spatial variability gets a value 1, 2 or 3 for scanners 1, 2 and 3, respectively. We used analysis of variance (ANOVA) and Akaike information criterion (AIC) to select the best model. If the ∆AIC was < 2 for several models, we chose the model with fewer parameters. The models tested were:

${G}_1=a+{b}_TT+{b}_RR+{c}_{R,i}+\varepsilon$ (model 1)

${G}_2=a+{b}_TT+{b}_RR+{c}_{S,j}+\varepsilon$ (model 2)

${G}_3=a+{b}_TT+{b}_MM+{b}_RR+{c}_{S,j}+\varepsilon$ (model 3)

${G}_4=a+{b}_TT+{b}_RR+{c}_{S,i}+{d}_{S,j}M+\varepsilon$ (model 4)

${G}_5=a+{b}_TT+{b}_RR+{c}_{RS, ij}+{d}_{RS, ij}M+\varepsilon$ (model 5)

where *a* is a fixed effect intercept, *b_T_*, *b_M_* and *b_R_* are fixed effect parameters related to soil temperature, soil moisture and root type, respectively. *c_R,i_* and *c_S,j_* are random effect intercepts related to root types *i* (0,1) or scanners *j* (1,2,3), respectively. *d_S,j_* is a random effect parameter related to soil moisture that varied between scanners *j* and *d_RS,jj_* a random effect parameter that accounts for the combined random effect of scanner and root type on soil moisture sensitivity. *ε* is model error.

All the statistical analyses were produced by R software (R Core Team; R version 3.5.3; RStudio version 1.2.1335) where we used ‘lme4’ package in the linear mixed-effect model analyses (Bates et al. 2015). *P* values were achieved from package ‘lmerTest’ (Kuznetsova et al. 2017), whereas *R*^2^ values were calculated by package ‘MuMIn’ (Bartoń 2018). Post hoc Tukey’s HSD tests were performed by ‘multcomp’ package (Hothorn et al. 2008).

### Comparison of belowground and aboveground growth phenology

We estimated the growth phenology of aboveground tree organs, such as shoots, needles, buds and secondary xylem using the ‘Carbon Allocation Sink Source Interaction’ (CASSIA) model (Schiestl-Aalto et al. 2015). CASSIA is a dynamic growth model that simulates the growth phenology and daily growth rates of tree organs (kg C day^−1^) based on environmental factors. It is constructed and parameterized at the measurement site and produces accurate estimates of aboveground growth (Schiestl-Aalto et al. 2015). We compared the relative growth rates of modeled aboveground organs with the measured root growth data of this study to examine the differences in the yearly growth pattern of the different organs. The root growth data were determined from the accumulated root surface area of the three scanners. Relative accumulated growth of organ *j* on day *i*, ${R}_{i,\,j}$(ϵ [0,1]) was calculated as:

(1) \begin{equation*} {R}_{i,j}=\frac{G_{i,j}}{G_{365,j}} \end{equation*}

where ${G}_{i,j}$is the absolute growth accumulation on day *i* (kg C) and ${G}_{365,\,j}$ the total growth at the end of the year. Furthermore, the relative growth rate of organ *j* on day *i*$({dR}_{i,j})$is

(2) \begin{equation*} {dR}_{i,j}={R}_{i,j}-{R}_{i-1,j} \end{equation*}

## Results

### Root characteristics

In total, 185 roots were traced, of which 68 were pioneer roots and 117 were fibrous roots. Table 1 presents the characteristics of different root types for the three scanners. The initiation time of the first root varied from May to August, and the cessation time of the last root varied from mid-October to end-October between different scanners (Table 1). Pioneer roots initiated earlier and ceased elongation later than fibrous roots. All roots had stopped elongation by 30 Oct (DOY 303). However, few pioneer roots were not completely suberized, which could be seen as the white color typical for the non-suberized parts of the roots. The average growing period of fibrous roots was 20–32 days shorter than that of pioneer roots in this study. Root characteristics such as root length and average growing period did not show clear differences between root types (Table 1). The fibrous roots characteristics (diameter, length) did not differ significantly (*P* > 0.05) between the three scanners (Table 1). The pioneer roots characteristics (diameter, length) of Scanner 1 and Scanner 2 were significantly (*P* < 0.05) thicker and longer than those of Scanner 3.

**Table 1:** Root growth characteristics in the three scanners. Initiation date is the first root appearance time, while cessation date means the time the last root ceased its growth. Values (for RDiam, RL, GP) are means with standard errors in parentheses. The differences of means were examined by two-way ANOVA, followed by Tukey’s post hoc test.

	Fibrous			Pioneer		
	Scanner 1	Scanner 2	Scanner 3	Scanner 1	Scanner 2	Scanner 3
No. roots	32	31	54	34	18	16
Initiation date^1^	6 Aug (218)	20 May (140)	13 Jul (194)	19 Jul (200)	7 May (127)	18 Jun (169)
Cessation date^2^	16 Oct (289)	22 Oct (295)	26 Oct (299)	30 Oct (303)	30 Oct (303)	21 Oct (294)
RDiam^3^	0.48^a^ (0.02)	0.58^a^ (0.02)	0.42^a^ (0.02)	1.23^c^ (0.08)	1.20^c^ (0.08)	0.80^b^ (0.04)
RL^4^	14.4^a^ (1.65)	13.0^a^ (1.24)	8.55^a^ (0.81)	52.6^b^ (7.49)	56.1^b^ (8.56)	23.4^a^ (4.96)
GP^5^	29^b^ (2)	18^ab^ (3)	15^a^ (1)	45^c^ (4)	50^c^ (9)	27^ab^ (6)

^1^Initiation date indicates the date of first appearance of root on the screen. Day of Year (DOY) numbers in the parenthesis after the date.

^2^Cessation date indicates the date of last root ceased.

^3^Root diameter (RDiam), unit is mm.

^4^Root length (RL), unit is mm.

^5^Average growing period length (GP), unit is day.

The lowercase letters indicate the statistical differences between each root type of each scanner with significance level of *P*$\le$ 0.05.

The mean soil temperatures at the initiation time (7 May, DOY 127) and cessation time (30 Oct, DOY 303) of pioneer roots were 5.7 and 3.9 °C, respectively (Figure 1, Table 1). For fibrous roots, the mean soil temperatures at the initiation time (20 May, DOY 140) and cessation time (26 Oct, DOY 299) were 7.9 and 5.7 °C, respectively (Figure 1, Table 1) i.e., The pioneer roots were able to grow in ca. 2 °C lower soil temperature than the fibrous roots both in the beginning and in the end of the growing period.

### Active root number

**Figure 3.**
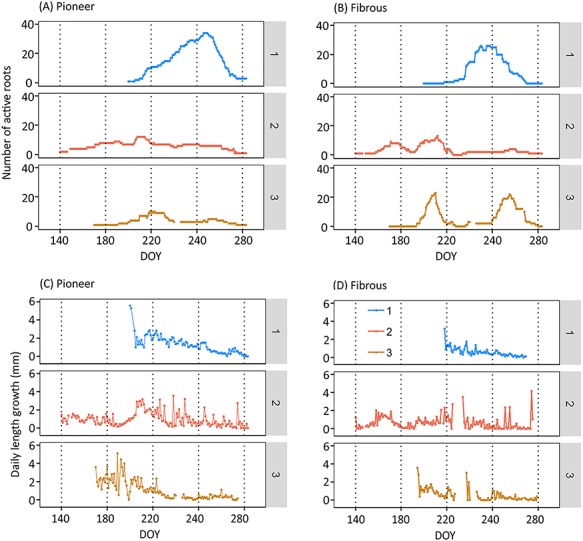
(A, B): Variation of number of active roots of different root types (pioneer, fibrous) during the growing season. (C, D): Variation of root elongation (mm day^−1^) of different root types (pioneer, fibrous). Note: The numbers 1, 2, 3 indicate the different scanners. The missing values of scanner 2 during DOY 226–233 (14–21 Aug) and scanner 3 during 16–24 Aug (DOY 228–236) were due to the absence of active fibrous roots. Other missing data were due to equipment problems: 27–28 May and 4 Aug (for scanner 2); 29 Aug–2 Sep and 14 Oct (for scanner 3). DOY stands for day of year, the sequential day number starting from 1 Jan 2018.

The number of active fibrous roots peaked 0–17 days before the peak of active pioneer roots (Figure 3A and B). All the flushes occurred during summer and early autumn when the temperature was the highest (Figure 1A). There were two obvious flushes of the active roots whereas other peaks were smaller: the first obvious flush happened for both Scanner 2 and 3 during 27 Jul–7 Aug (DOY 208–219); and the second flush for both Scanner 1 and 3 during the early autumn of 12 Sep–2 Oct (DOY 255–275) (Figure 3A and B).

### Influence of soil temperature, moisture and root type on root growth rate

Mixed model 1 showed a significant positive effect of temperature (parameter *b_T_)* on growth rate (*P* < 2e−16). Furthermore, the second mixed model (model 2) showed a significant fixed effect of root type (parameter *b_R_*) on growth rate (*P* < 2e−15) and also the random effect related to spatial variability (parameter *c_S_*) was significant (*P* < 3e−11). Adding soil moisture as a fixed effect (model 3) did not improve the model (Table 2). However, adding a scanner-dependent soil moisture effect (parameter *d_S,j_* in model 4) significantly improved the model (Table 2). Finally, we added a random parameter (*d_RS,ij_* model 5) that accounted for the combined effect of scanner and root type on soil moisture sensitivity of root growth. As model 5 was better than model 4 based on both AIC and ANOVA comparisons, soil moisture effect on root growth rate proved to vary both spatially and between root types (Table 2).

**Table 2:** Comparisons of the linear mixed models.

Compared models	ΔAIC	*P*
Models 1 and 2	44	<2e−16^*^
Models 2 and 3	0.4	>0.1
Models 2 and 4	18	<6e−6^*^
Models 4 and 5	8	<0.005^*^

^*^Probability of the higher order model of being better than the previous model, *P* indicates *P* values in ANOVA comparison; ΔAIC indicates difference of AIC values.

Model 5 showed a positive temperature effect on growth rate and a higher overall growth rate of pioneer than fibrous roots (Table 3). The effect of soil moisture was more complicated: the effect of soil moisture was positive on fibrous roots, whereas on pioneer roots, the effect was negative in Scanners 1 and 2 and positive in Scanner 3 (slope parameter *d_RS_* in Table 3). The measured root length growth values with mixed model 5 fitted values by different scanners and different root types could be found in Figure 5.

**Table 3:** Values of the parameters of mixed model 5 for different root types and scanners.

Parameter	Scanner	Fibrous	Pioneer
*c_RS_*	1	−0.07	0.86
	2	−0.28	0.13
	3	−0.98	−1.46
*d_RS_*	1	0.27	−4.06
	2	1.31	−0.57
	3	4.76	6.98

Note: The common parameters are *a* = −0.84, *b_T_* = 0.13, *b_R_* = 0.32. *a* is a fixed effect intercept, *b_T_*, *b_R_* are fixed effect parameters related to soil temperature and root type, respectively. *c_RS_* indicates random effect intercept related to root types (fibrous, pioneer) or scanners (1,2,3). *d_RS_* is a random effect parameter related to soil moisture that varied between scanners and root type.

**Figure 4.**
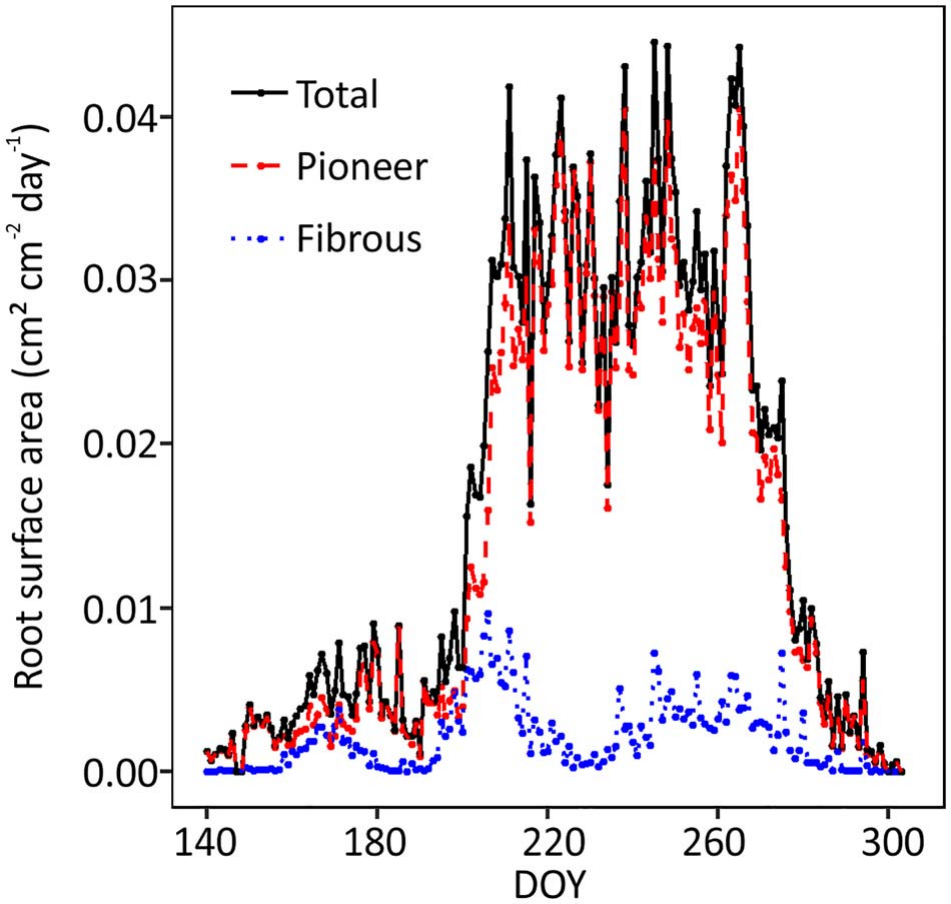
Root surface area growth variations during year 2018. The surface area growth was determined from the accumulated root surface area growth data of three scanners. DOY stands for day of year, the sequential day number starting from 1 Jan 2018.

**Figure 5.**
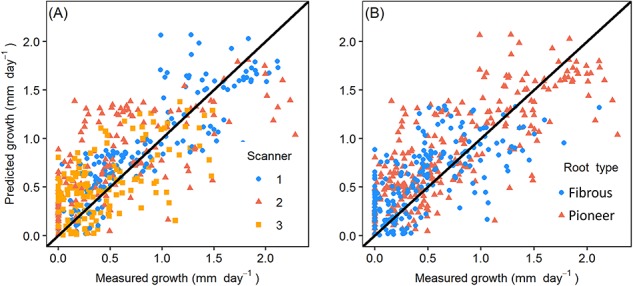
The measured root length growth with model fitted values of (A) different scanners (B) different root types. *R*^2^*m* = 0.38, *R*^2^*c* = 0.50, *R*^2^*m* described the proportion of variance explained by fixed effects. *R*^2^*c* represented the proportion of variance explained by both fixed and random effects.

In August, a summer drought occurred in Southern Finland when the lowest soil moisture was ca. 0.10 m^3^ m^−3^. The drought period lasted for about 1 month and the soil temperature was the highest during that period (Figure 1). Fibrous roots suffered at the beginning of the drought, unlike pioneer roots, which were independent of soil moisture (Figures 3 and 4), also convinced in the mixed model 5. There were no fibrous root extensions on two out of three scanners (DOY 226–236) (Figure 3B and D), and their surface area growth rate clearly decreased at the beginning of the summer drought (ca. DOY 220) (Figures 1 and 5). The pioneer roots accounted for 87% of total root surface area (Figure 4), and there was no notable drop in their surface area growth (Figure 4).

**Figure 6.**
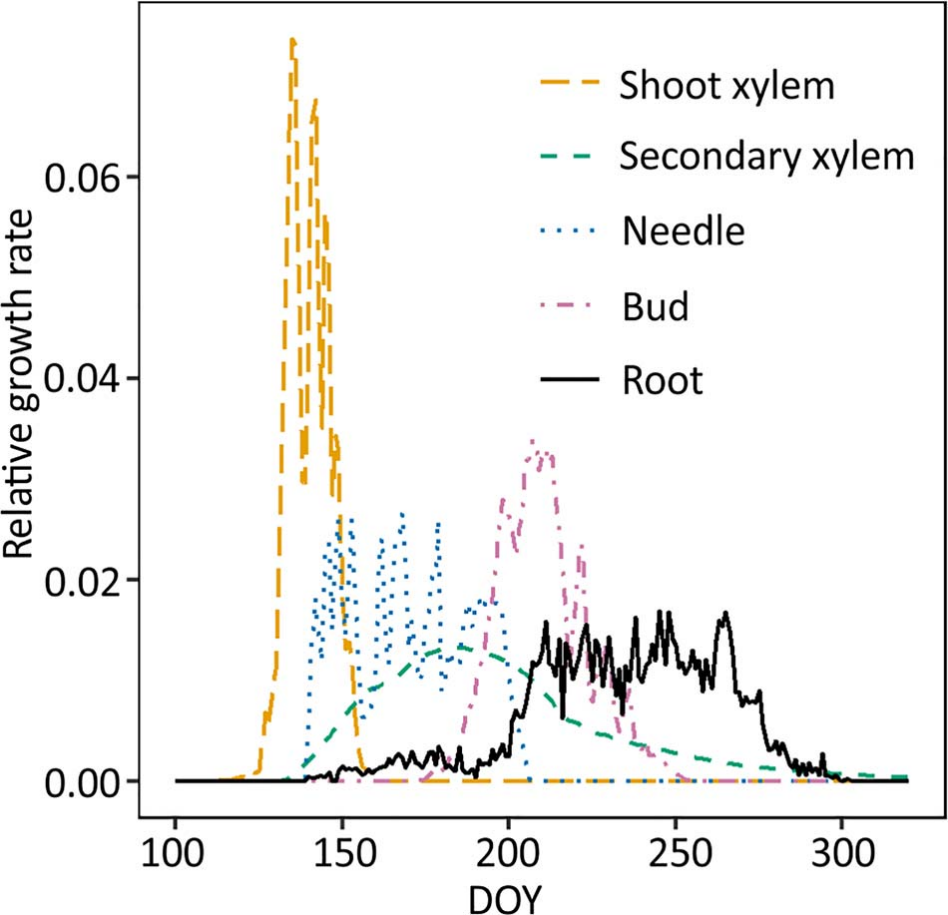
Estimated above- and belowground phenology of scots pine. Aboveground growth was modeled by CASSIA (Schiestl-Aalto et al. 2015), root growth (black solid line) was measured in this study. Note that the y-axis was relative growth, i.e., it only described the timing of growth, not the carbon used for growth. Integral of each line = 1.

### Above- and belowground growth phenology

The timing of aboveground shoot xylem, secondary xylem, needle and bud growth was estimated using the dynamic growth model CASSIA (Schiestl-Aalto et al. 2015) with the abiotic data from SMEARII station (Figure 6). Primary growth, i.e., shoot growth, began first in the spring and ceased earliest, by the end of June. Shoot growth onset was followed by needle and secondary growth onset and their growth period was longer than that of shoots. Secondary growth continued until late summer as cell wall formation of new cells is a long process. Bud growth took place in July–August. Observed roots began intensive growth concurrently with needle growth cessation and when the majority of all aboveground growth had occurred.

## Discussion

### Differences in pioneer and fibrous root growth

We observed significant root morphology differences between pioneer and fibrous roots of Scots pines growing in the southern boreal zone (Table 1, Figure 2). As hypothesized, the elongation rate of fibrous roots was more sensitive to severe weather conditions (drought and low temperature) compared to the pioneer roots. Growth differences between the two root types may be due to differences between root anatomical structures and fungal colonization (Zadworny and Eissenstat 2011). Pioneer roots tend to have higher construction costs with more primary xylem poles and larger tracheid diameters compared to fibrous roots (Guo et al. 2004, Bagniewska-Zadworna et al. 2012). Pioneer roots tend to live longer, branch more intensively, provide larger transport capacity and have better adaptability to biotic and abiotic challenges than fibrous roots. In contrast, they have limited absorption ability since secondary development happens frequently in pioneer roots (Zadworny and Eissenstat 2011, Polverigiani et al. 2011, Bagniewska-Zadworna et al. 2012). Moreover, the pioneer roots with secondary root xylem development can transport nutrients and water to aboveground components. So far, we do not know of any reports about differences of pioneer and fibrous root growth phenology on boreal EcM species such as conifers.

### Soil temperature and root elongation

Our results gave support to our second hypothesis that the pioneer roots were more tolerant to lower soil temperature than the fibrous roots as the threshold temperature of pioneer and fibrous growth cessation was 5.7 and 3.9 °C, respectively. In line with our results, Alvarez-Uria and Körner (2007) indicated that the critical temperature below which root growth of Scots pine significantly inhibited was 4–6 °C, whereas Wang et al. (2018) indicated that coniferous roots of mixed forests in general were able to continue growing above temperature threshold of 0 °C. In addition, we found a significant positive relationship between root elongation rate and soil temperature as also suggested by other studies (Pregitzer et al. 2000, Iivonen et al. 2001, Steinaker and Wilson 2008, Blume-Werry et al. 2016) for woody species if there are no other limitations (e.g., soil moisture and nutrients); however, none of these studies have observed root growth patterns by daily frequency. Furthermore, the effect of soil warming on root elongation rate can be further promoted by the increased rate of soil organic matter decomposition and induced N mineralization, which promotes root extension (Pregitzer et al. 2000).

### Soil moisture and root elongation

There were both positive and negative correlations between root growth rate and soil moisture among scanners most probably indicating that the mean soil moisture of the stand used in the analysis did not represent the moisture environment next to each scanner. First of all, the negative correlation of root growth with soil moisture may have been caused by the negative covariance between temperature and moisture (Spearman’s rho = −0.54, *P* < 0.0001). It is well known that the soil–water-holding capacity as well as the distribution of roots varies spatially resulting in small-scaled spatial variation in soil moisture. Thus, we cannot conclude that soil moisture affected root growth rate both negatively and positively but the results highlighted that part of the roots might grow in suitable conditions even though most of the stand would suffer from drought. Nevertheless, our study showed that the growth of pioneer roots was not as clearly influenced by drought as that of fibrous roots, which was clearly slow at the beginning of the summer dry period (Figure 4). Furthermore, the possible heterogeneity of soil moisture also could affect the distributions of pioneer and fibrous roots. Woody species tend to spread their pioneer roots deeper or horizontally away from the stem whenever drought or low temperature occurs in order to find a more humid and warmer location. Whenever a pioneer root grows to a preferred location with humid and nutrient-rich soil, fibrous roots will branch intensively from the pioneer root to absorb water and nutrients. Fine roots, as the most distal part of a tree have been observed shredded during drought in forests, which according to the ‘cost-benefit’ theory maximizes the efficiency of nutrient acquisition as the root system evaluates the benefit and cost of building new roots and shredding old roots (Eissenstat and Yanai 2002, Chenlemuge et al. 2013). Moreover, Kotowska et al. (2015) stated that in line with a phenomenon called ‘hydraulic segmentation’ which is based on a concept from Zimmermann’s segmentation hypothesis on aboveground leaves (Tyree and Zimmermann 2002), the death of distal fine roots (fibrous roots) could protect coarse roots from a reverse water flow from fibrous root to the drier soil. Our results are in correspondence with a study on olive species showing that fibrous roots suffered physiologically from deficient soil moisture, whereas pioneer roots, with higher tissue density and suberin content, had stronger growth plasticity for the drought (Polverigiani et al. 2011).

### Timing of the root growth in relation to the aboveground tree growth

Our study shows that the initiation time of the first root (7 May) occurred simultaneously with shoot growth initiation (Figure 6). Although the initiation time was the same for above- and belowground growth, the intensive growing time and cessation time of aboveground organs were earlier than those of root growth (Figures 4 and 6). This is in line with a review of boreal, arctic and temperate biomes in which shoot growth was suggested to be in asynchrony with root growth (Abramoff and Finzi 2015). Similar kinds of results about growth of roots of woody species peaking and ceasing later than the growth of shoots have been obtained also in several other studies (Steinaker et al. 2010, Du and Fang 2014, Abramoff and Finzi 2015, Blume-Werry et al. 2016). In general, root and shoot growth have a greater tendency to be inconsistent in forests but consistent in grassland and tundra that have plant species with smaller aboveground biomass (Steinaker and Wilson 2008, Steinaker et al. 2010).

There are several empirically confirmed explanations about this time lag between aboveground growth and root growth based on both exogenous and endogenous factors. First, the air temperature increases and decreases more quickly than the soil temperature (Figure 1A) as the high specific heat capacity of soil can keep the temperature suitable for root growth even after the senescence of the aboveground leaves (Blume-Werry et al. 2016). Second, the carbohydrates reserved in twigs are stimulated by the warm air to support the growth of leaves in early spring to fulfill the photosynthesis process (Landhäusser and Lieffers 2003), while the roots cannot grow much since the shoots consume the majority of the photosynthates. After the aboveground growth is almost completed, the photoassimilates will be allocated to belowground to build root structures, which absorb nutrients and water from the soil (Sloan and Jacobs 2008, Abramoff and Finzi 2015). Lastly, on the physiological and molecular levels, several hormones like auxin, gibberellin and brassinosteroid may regulate root and shoot growth as the main mechanism of down-regulation of root growth (Depuydt and Hardtke 2011, Leyser 2018). However, the mechanisms of how the hormones are distributed inside plants are still not clear. All these explanations would mean that even though growth of roots and shoots is not synchronized they may not be independent of each other.

### The applicability of the scanner method

So far, methods to study root growth phenology have been limited. Here, the automatic measurements by the scanners required only a little labor during the measurements. We analyzed the images in a traditional manual way, but Nakano et al. (2012) have developed an automatic add-in to track root growth dynamics with results similar to manual calculations. With the development of tracking software, root study by scanners may be even less laborious in the future. However, the scanner method has also some flaws. It needs power and cable connections in the study fields. Furthermore, we suspect the time interval between installation and measurement may be a minor reason to affect the initiation time of the root and therefore, if possible, the recovery time before the measurements could be longer in future measurements. A suggestion of time lag before the first measurement was about 6–12 months after installation, but some studies have started to collect images immediately or only a few weeks after installation (Johnson et al. 2001). Even with these deficiencies, this technique is a very promising method for phenology and growth rate measurements. To our knowledge, there are very few series of daily root growth from boreal forests. With more variable ranges of weather changes in the future, usage of scanner method would enable more accurate determination of the drivers of root growth than has been so far possible.

## Conclusions

Scots pine root growth phenology was mainly driven by temperature in boreal forests. Furthermore, the soil moisture had a variable effect on the root length growth indicating spatial variation in the soil. Pioneer roots grew faster and were thicker than fibrous roots and they were more adaptable to severe weather conditions, such as low temperature and drought. By comparing the surface area growth of both types of roots, the pioneer roots were not affected by the summer drought, whereas the fibrous roots suffered from the soil moisture deficit at the beginning of the drought period. Root growth phenology was not synchronized with the growth of aboveground tree components (e.g., shoot, secondary xylem, needle, bud), the root growth peaked and ceased later than growth of aboveground organs. Our results suggest that besides considering the differences of growth phenology of above- and belowground vegetation components, whole tree growth models could benefit from separating the roots into different types, for example into pioneer and fibrous roots.

## Data and materials availability

Images and all other data are available from DOI: 10.5281/zenodo.3531606.

## Supplementary Material

Figure_S1_tpz131
