# Supplementary figures and images for: Temperature and moisture dependence of daily growth of Scots pine (Pinus sylvestris L.) roots in Southern Finland
Source: Tree Physiol. 2019 Dec 20;40(2):272–83. doi: 10.1093/treephys/tpz131 (PMC7048678; doi:10.1093/treephys/tpz131)

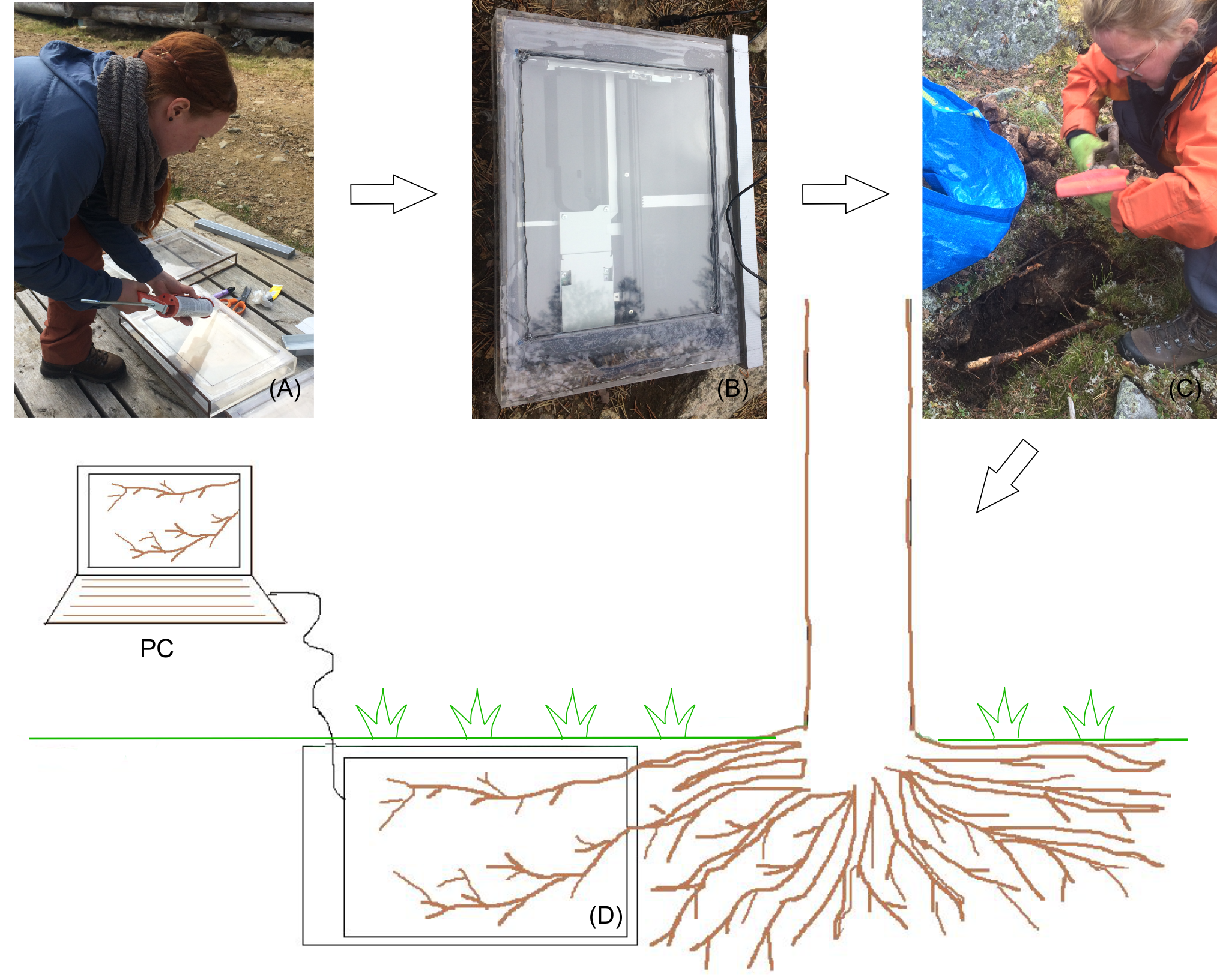

Supplement: Figure_S1_tpz131 [file figure_s1_tpz131.png]
